# Mechanical Properties of High-Volume Fly Ash Strain Hardening Cementitious Composite (HVFA-SHCC) for Structural Application

**DOI:** 10.3390/ma12162607

**Published:** 2019-08-16

**Authors:** Chenhua Jin, Chang Wu, Chengcheng Feng, Qingfang Zhang, Ziheng Shangguan, Zuanfeng Pan, Shaoping Meng

**Affiliations:** 1 School of Civil Engineering, Southeast University, Nanjing 211189, China; 2 School of Public Administration, National Research Center for Resettlement, Hohai University, Nanjing 211100, China; 3 College of Civil Engineering, Tongji University, Shanghai 200092, China

**Keywords:** strain hardening cementitious composite, fly ash, micromechanics, particle size distribution, tensile strain, compressive strength

## Abstract

Strain-hardening cementitious composite (SHCC) is a kind of construction material that exhibits multiple cracking and strain-hardening behaviors. The partial replacement of cement with fly ash is beneficial to the formation of the tensile strain-hardening property of SHCC, the increase of environmental greenness, and the decrease of hydration heat, as well as the material cost. This study aimed to develop a sustainable construction material using a high dosage of fly ash (no less than 70% of the binder material by weight). Based on the micromechanics analysis and particle size distribution (PSD) optimization, six mixes with different fly ash to cement ratios (2.4–4.4) were designed. The mechanical properties of the developed high-volume fly ash SHCCs (HVFA-SHCCs) were investigated through tensile tests, compressive tests, and flexural tests. Test results showed that all specimens exhibited multiple cracking and strain-hardening behaviors under tension or bending, and the compressive strength of the designed mixes exceeded 30MPa at 28 days, which is suitable for structural applications. Fly ash proved to be beneficial in the improvement of tensile and flexural ductility, but an extremely high volume of fly ash can provide only limited improvement. The HVFA-SHCC mix FA3.2 (with fly ash to binder ratio of about 76% by weight) designed in this study is suggested for structural applications.

## 1. Introduction

Coal-burning thermal power stations are still the major source providing power globally. It is known that fly ash (FA) is a principal byproduct of coal combustion in thermal power plants. As a result, large quantities of fly ash have been produced by these coal-fired power plants in recent years. Reports indicate that the top three countries that produced the most fly ash are China (around 600 million tons), India (around 220 million tons), and the United States (around 130 million tons) [1,2]. However, the reuse rates of fly ash attributed to these countries are only approximately 70%, 60%, and 50%, respectively [1,2], while large amounts of the remaining fly ash are disposed of in landfills, ash ponds, or mine voids. With these disposal methods, there are potential risks of air pollution and groundwater contamination due to the leakage of fly ash [3,4,5]. Fortunately, FA has been classified as a non-hazardous waste by the Environmental Protection Agency of the United States [6]. Fly ash has also been suggested to be useful in beneficial applications, such as in agriculture, hazardous waste immobilization, and construction [7]. A major application of fly ash in construction is to use it as a pozzolanic mineral admixture in concrete. The recycled fly ash is added to the mixture to partially replace cement as a binder material. Replacement of cement with fly ash can not only increase the environmental greenness, but also can decrease the hydration heat, and meanwhile, the material cost can be reduced as well [8]. Moreover, research investigations [4,9] have reported that the addition of fly ash confers the advantages of enhanced durability and reduced drying shrinkage. Based on the morphological and micro-aggregate effects, the small particle size and smooth spherical shape of un-hydrated fly ash particles result in better workability, higher compactness in the interfacial transition zone, and a finer pore structure in the system [10,11,12,13]. Since fly ash does not have its own hydraulic property, the use of fly ash in large quantities can weaken the neutralization and reduce the strength. However, desirable mechanical and durability properties of high-volume fly ash (HVFA) concrete have been achieved by carefully designing the mix proportions and utilizing super-plasticizers (SPs) [14,15]. Another important application of FA is to produce a kind of high-performance fiber reinforced cement composite (HPFRCC), known as engineered cementitious composites (ECCs), developed by Li et al. [16,17,18]. ECCs generally consist of cement, fly ash, fine aggregates, water, SPs, and short fibers less than 2% by volume. ECCs exhibit distributed multiple fine cracks, with crack width usually controlled to be less than 100 μm and strain-hardening behavior with the ultimate tensile strain over 3.0%. To emphasize the unique tensile strain-hardening characteristic, the International Union of Laboratories and Experts in Construction Materials, Systems and Structures (RILEM) TC HFC (technical committee of high performance fiber reinforced cementitious composites) named this class of materials strain -hardening cementitious composites (SHCC) [19,20]. It has been proven that the addition of FA can change the bond between matrix and fibers, contributing to the formation of multiple cracks and the strain-hardening property [18,19]. In terms of durability, it was more favorable for cement-based composites to have more cracks but with small width, rather than fewer cracks but with large width. Szeląg [21,22] confirmed this result with the evaluation of crack patterns of cement paste containing polypropylene fibers under thermal loads. Li [19] also indicated that ECC characteristics of multiple cracks with fine crack width were favorable to its durability. A high volume of fly ash (no less than 70% of the binder material) was applied in the development of SHCC for structural applications. Six mixes with different fly ash/cement ratios were designed based on the micromechanics analysis and particle size distribution (PSD) optimization to fulfil the objective of tensile strain-hardening behavior and target compressive strength of 30 MPa. Tensile tests, compressive tests, and flexural tests were conducted in this study to investigate the influence of fly ash content on the mechanical properties of designed HVFA-SHCCs.

## 2. Material Design 

### 2.1. Objective

Three goals were set as requirements for the mix design of the HVFA-SHCCs: (1) the composites must exhibit tensile strain-hardening behavior and multiple cracking behavior; (2) the fresh mixture should show acceptable flowability, homogeneity, and fiber dispersion; and (3) the HVFA-SHCC materials are designed for structural application, and thus should have reasonable compressive strength.

To achieve the first goal, micromechanics-based analysis was done, and two criteria, known as strength and energy criteria proposed by Li [8,9], needed to be satisfied. The tensile strain capacity of the developed material was expected to be greater than 2%. Particle size distribution of dry materials for mixing could be easily optimized to meet the second requirement. The mixed HVFA-SHCC was expected to possess compressive strength greater than 30 MPa according to compressive tests, which meets most of the minimum requirements with respect to application in different structural contexts, according to the Chinese standard for design of concrete structures (GB50010-2010) [23].

### 2.2. Micromechanics-Based Analysis

To ensure the characteristics of tensile strain hardening and multiple cracking, Li [24,25] indicated that two conditions must be satisfied based on the micromechanical model. These two conditions are categorized in a strength criterion, called the first cracking stress criterion, and an energy criterion, called the steady state cracking criterion, and the expressions are as follows:

(1) Strength criterion

(1) $$\sigma_{fc}\leq \sigma_{0}$$

(2) Energy criterion

(2) $${J^{\prime }}_{b}\equiv \sigma_{0}\delta_{0}-\int_{0}^{\delta_{0}}\sigma \left(\delta \right)d\delta \geq J_{tip}\approx \frac{K_{m}^{2}}{E_{m}}$$

where *σ_fc_* and *σ*_0_ are the cracking strength and maximum fiber-bridging strength on a crack plane; *J*_b_′ is the complementary energy calculated from bridging stress-crack opening (*σ*-*δ*) curve and *δ*_0_ is the crack opening corresponding to *σ*_0_ in the fiber-bridging relationship *σ*(*δ*); *J_tip_* is the crack tip toughness, which can be approximately obtained from the matrix toughness and elastic modulus if fiber volume fraction is less than 5%; and *K_m_* and *E_m_* are the matrix fracture toughness and Young’s modulus, respectively.

The strength criterion requires that the first cracking strength must be less than the maximum fiber-bridging stress on each crack plane, otherwise, the crack plane will lose its tensile capacity as a result of fiber rupture or pull-out. The energy criterion implies that the crack tip toughness should be less than the complementary energy, otherwise, steady-state cracking cannot be formed, which will result in local failure in the middle of the crack plane, and, consequently, multiple cracking characteristics can hardly be exhibited. If the complementary energy *J*_b_′ is insufficient or the crack tip toughness *J_tip_* is too high, the energy criterion will be hardly satisfied.

To quantify the complementary energy, the *σ*-*δ* relationship should be determined. Li et al. [16,26] proposed a fiber-bridging model specifically for randomly distributed short-fiber-reinforced cement-based composites on the basis of micromechanics and fracture mechanics. Lin et al. [25] then proposed a modified fiber-bridging model for calculating the relationship between fiber-bridging stress and crack opening width, with which the effects of fiber–matrix interface bonding action and fiber rupture could be considered. Yang et al. [27] further modified the fiber-bridging model with the consideration of two-way fiber pullout, matrix micro-spalling, and the Cook–Gordon effect. Yang’s model is coded with MATLAB and was employed in this study to predict the bridging behavior of the polyvinyl alcohol (PVA) fiber which was used in the development of the HVFA-SHCCs.

To assure that the material can steadily exhibit strain hardening and multiple cracking, Kanda and Li [28] suggested *σ*_0_/*σ_fc_* and *J*_b_’/*J_tip_* as two performance indicators of pseudo strain hardening for tailoring the mix. According to the aforementioned criteria for pseudo strain hardening, the values of both *σ*_0_/*σ_fc_* and *J*_b_’/*J_tip_* must be greater than 1.0. For SHCC reinforced with PVA fibers, the requirement of *J*_b_’/*J_tip_* > 3 and *σ*_0_/*σ_fc_* > 1.45 should be met, as suggested by Kanda and Li [28].

**Figure 1:**
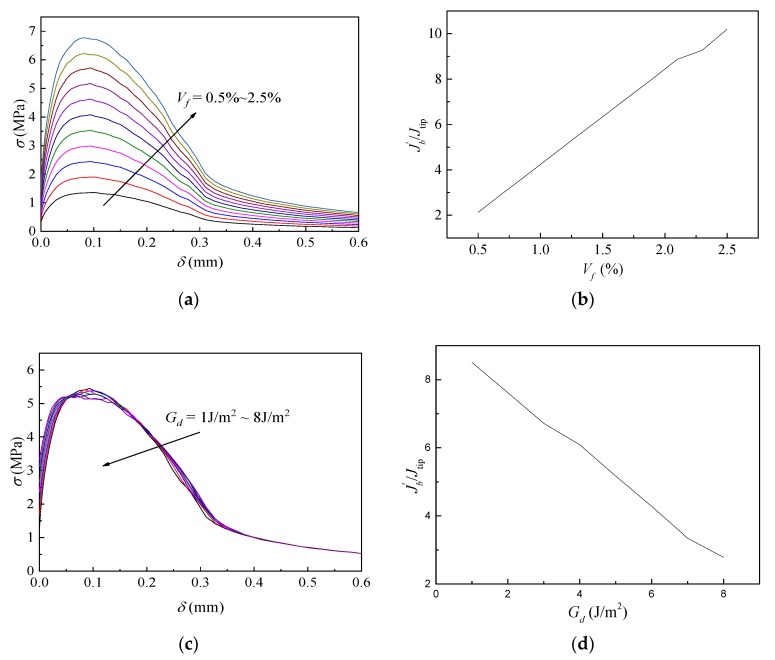

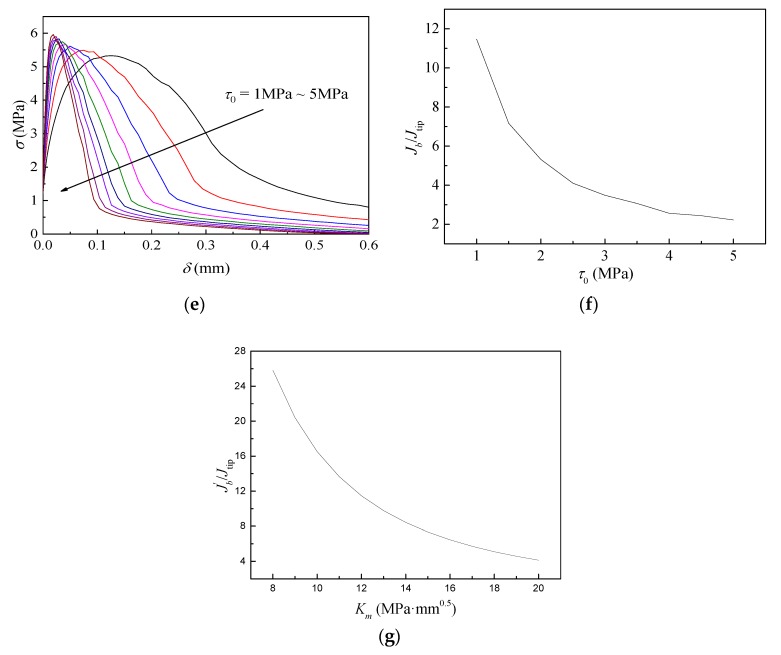
Effects of the analytical parameters on the fiber bridging stress-crack opening width (*σ-δ*) curve and performance indicator (*J_b_*′/*J_tip_*). (**a**) Effect of fiber volume fraction (*V_f_*) on *σ-δ* curve; (**b**) *V_f_* vs. *J_b_*′/*J_tip_*; (**c**) effect of chemical bond (*G_d_*) on *σ-δ* curve; (**d**) *G_d_* vs. *J_b_*′/*J_tip_*; (**e**) effect of frictional bond (*τ*_0_) on *σ-δ* curve; (**f**) *τ*_0_ vs. *J_b_*′/*J_tip_*; (**g**) fracture toughness of matrix (*K_m_*) vs. *J_b_*′/*J_tip_*.

The characteristics of SHCC are mainly related to the properties of the reinforcing fiber, the matrix, and their interface. Based on the fiber-bridging model developed by Yang et al. [27], parametric analyses were conducted to study the influence of the volume fraction of fiber (*V_f_*), chemical and frictional bond of the fiber–matrix interface (*G_d_* and *τ*_0_), and the fracture toughness of the matrix (*K_m_*), which can be regarded as guidance for the designing of SHCC materials. The basic parameters used in the model refer to the values provided in Reference [29] and are shown in Table 1. The effects of the variables on the *σ-δ* relationship curve and performance indicator *J*_b_’/*J_tip_* are shown in Figure 1.

Figure 1a,b indicate that the maximum fiber-bridging strength *σ*_0_ increases linearly with the increase of *V_f_*, while the corresponding crack opening *δ*_0_ stayed almost the same. Therefore, the energy performance indicator *J*_b_’/*J_tip_* also increased linearly. In terms of the chemical bond *G_d_*, Figure 1c,d show that *σ*_0_ did not change significantly, but *δ*_0_ decreased slightly and *J*_b_’/*J_tip_* also decreased linearly, with increasing *G_d_*. Figure 1e,f demonstrate that with the increase of *τ*_0_, bridging stress *σ*_0_ increased slightly, however, *δ*_0_ decreased significantly, and consequently, *J*_b_’/*J_tip_* decreased dramatically at the beginning and gently in the end. The fracture toughness of matrix *K_m_* also affected the performance indicator *J*_b_’/*J_tip_*. From Figure 1g, it can be seen that the performance indicator decreased parabolically with increasing *K_m_*.

Based on the analysis, a higher volume of fiber can provide more complementary energy, however, excessive fibers may negatively affect workability and fiber dispersion. Li [19] suggested that the optimized fiber volume fraction in SHCC material is around 2%, which can simultaneously satisfy the requirement of the energy criterion and perform well in workability. Chemical and frictional bonds should not be too strong, otherwise, the complementary energy will be too low to meet the energy criterion for SHCCs. The hydrophilic characteristics of PVA fiber may lead to excessive chemical and frictional bonds, and, consequently, the fibers are vulnerable to rupture when the composite is subjected to tension. However, the bonds between fiber and matrix can be improved by adjusting the water to binder ratio and fly ash to cement ratio. Properly increasing the water to binder ratio and fly ash to cement ratio can decline both the bond strength of the interface and the fracture toughness of the matrix, which is beneficial to the formation of strain hardening and multiple cracking. It should be also noted that increasing the water to binder ratio will reduce the compressive strength, and therefore the water content should be properly controlled.

### 2.3. Particle Size Distribution (PSD) Optimization

In order to produce a strain-hardening composite with the expected flowability, particle packing theory was used as a tool to guide the mix design. It is confirmed that there is a positive relationship between rheological properties and the packing density of the concrete mix: the better the packing, the more water is available to act as a lubricant for the solids, and the better the fluidity [30,31]. A continuous grading of all solids (aggregate and powders) will result in a better workability and stability of the concrete mix [30]. Moreover, materials designed with this methodology can be processed using nearly any equipment, not only the high-energy, force-based mixers commonly used in academic research laboratories or precast concrete plants [32].

For regular concrete, the Fuller curve is always selected as a target curve for optimizing the packing density of the solid material. However, for modern concretes, such as high strength concrete (HSC), high-performance fiber-reinforced cementitious composite (HPFRCC), and self-compacting concrete (SCC), this Fuller curve is less suited [30]. The modified Andreasen and Andersen (A&A) curve [33] has proven successful for material design of ceramics [32], and many researchers have selected it as a target curve for designing the mixture of SCCs [31] and fiber-reinforced SCCs [34], with reasonable results. In this mix design method, the modified Andreasen and Andersen PSD model, as shown in Equation (3), was also utilized as the optimal grading curve function, and a number of mixtures were designed with PSD curves as close to the optimal curve as possible.

(3) $$P(D)=\frac{D^{q}-D_{\min}^{q}}{D_{\max}^{q}-D_{\min}^{q}}\times 100$$

where *P* is the cumulative fraction of particles that are finer than a certain diameter of *D*; *D*_min_ and *D*_max_ are the minimum and maximum particle size in the distribution, respectively; and *q* is a parameter with a value between 0 and 1, called the distribution modulus.

For the distribution modulus *q*, different scholars give different suggestions. Based on Funk and Dinger’s research [33], the distribution modulus is recommended to be 0.37, while Hunger [35] suggested a value of 0.25 for the design of self-compacting concrete. Yu et al. [36] think that a smaller distribution modulus will lead to increased usage of fine materials and thus result in an increase of water requirement. Hüsken [37] thinks that the compressive strength of the mixed material will decrease with the increase of *q*. In this study, 0.25 was used as the distribution modulus in the preliminary mix design.

**Figure 2:**
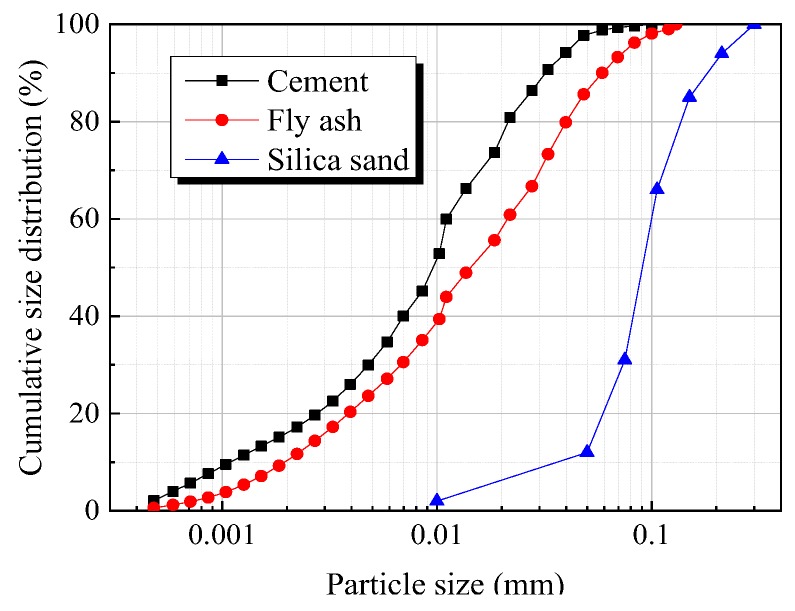
Particle size distribution of raw materials.

**Figure 3:**
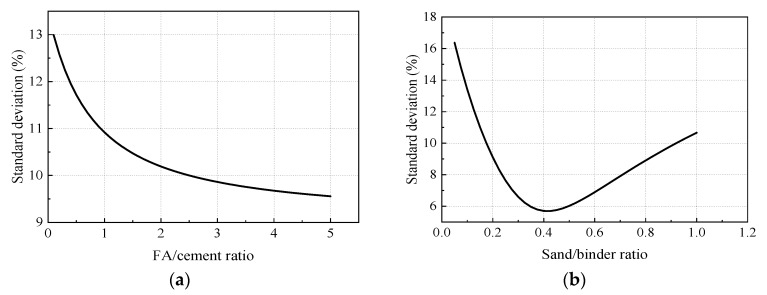
Effect of fly ash (FA) to cement ratio and sand to binder ratio on the standard deviation between the designed PSD curve and the optimal grading curve. (**a**) Effect of FA to cement ratio; (**b**) effect of sand to binder ratio.

The particle size distributions of all the dry materials (cement, fly ash, and silica sand) applied in this study are shown in Figure 2. Standard deviation of PSD curve of the designed mixture compared to optimal grading curve calculated from the A&A model was considered as an index to evaluate how closely the designed PSD curve fit to the optimal curve. Figure 3a,b shows the effect of FA to cement ratio and sand to binder ratio on the standard deviation between the designed PSD curve and optimal grading curve, respectively. It is illustrated from Figure 3 that with the increase of FA to cement ratio, the standard deviation reduced parabolically, which indicates that the use of fly ash improved the packing density. The standard deviation was less than 10% and reduced less significantly when the FA to cement ratio exceeded 2.4, while the standard deviation decreased initially and then rose with increase of the sand to binder ratio. The minimal standard deviation appeared when the sand to binder ratio stayed at around 0.4.

## 3. Experiments

### 3.1. Mix Proportions

**Figure 4:**
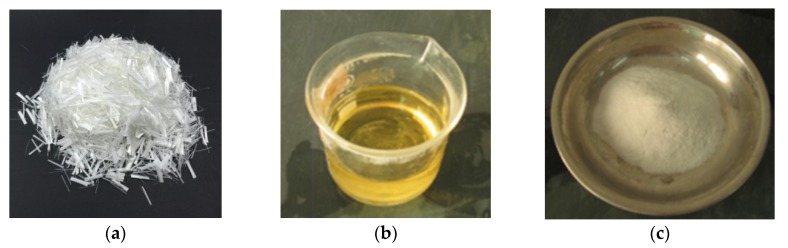

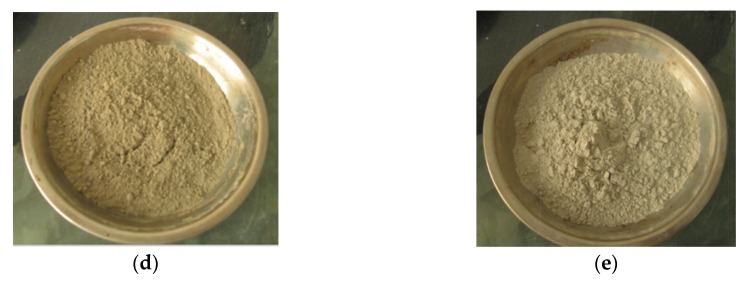
Raw materials used in the preparation of the HVFA-SHCC samples. (**a**) polyvinyl alcohol (PVA) fiber; (**b**) superplasticizer (SP); (**c**) silica sand; (**d**) fly ash; (**e**) cement.

Based on the aforementioned micromechanics-based analysis and PSD optimization, six HVFA-SHCCs with different FA to cement ratios were designed to investigate their mechanical properties, as shown in Table 2. The matrix used in this study consisted of Portland cement (P.II.42.5), silica sand (with average and maximum sizes of 110 μm and 300 μm respectively), and Grade I fly ash (supplied by Nanjing Thermal Power Plant). The chemical compositions of the cement and FA as provided by the manufacturers are listed in Table 3. The fiber used in this experimental study was Kuraray K-II REC15 PVA fiber, and its mechanical properties are shown in Table 4. A polycarboxylic-type superplasticizer (SP) was added as a water reducer. The raw materials used in this study are exhibited in Figure 4.

The mixing process was as follows: all the prepared dry materials, including cement, sand, and fly ash, were first mixed at a low speed for approximately 1.5 min. After the dry materials were fully mixed, water and SP were then added in sequence and mixed for another 3 min. Once a steady mixture with reasonable flowability was reached, PVA fibers were then slowly added into the fresh mixture while it was kept mixing until all fibers were adequately distributed. The whole mixing procedure typically took about 10 min.

### 3.2. Uniaxial Tensile Test

#### 3.2.1. Test Preparation for Uniaxial Tensile Test

**Figure 5:**
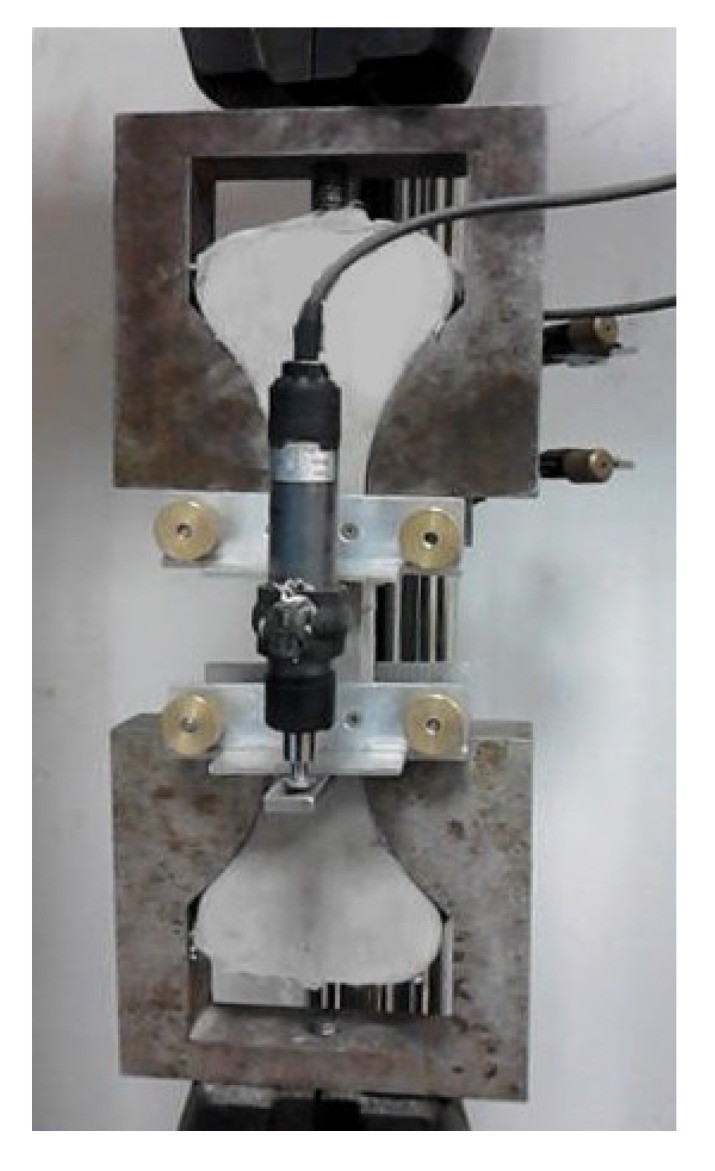
Test setup (tensile test system, Xinsansi, Shenzhen, China) for direct tension

The direct tensile test is a basic test that can best reflect the multiple cracking and strain-hardening properties of SHCCs. Compositions F2.4, F3.2, and F4.4 were selected to be tested in this study. Uniaxial tensile testing was performed with a dumbbell-shaped specimen with a cross section of 30 mm × 30 mm in the middle part. At least six dumbbell specimens were prepared for each mix group. The molds were removed after 24 hours, and then the specimens were cured in a standard curing room at a temperature of 23 ± 2 °C and relative humidity of 95% ± 5% for 28 days. The specimens were tested using a 30 kN capacity hydraulic servo loading system with displacement control, and the loading rate was 0.2 mm/min. Two linear variable differential transformers (LVDTs) were placed at the two sides of each specimen through a self-manufactured aluminum alloy frame to measure the displacement within a gauge length of 80 mm in the middle part of each specimen, as shown in Figure 5. The test stopped when the specimens entered the tension-softening stage due to the development of main cracks.

#### 3.2.2. Test Results of Uniaxial Tensile Test

**Figure 6:**
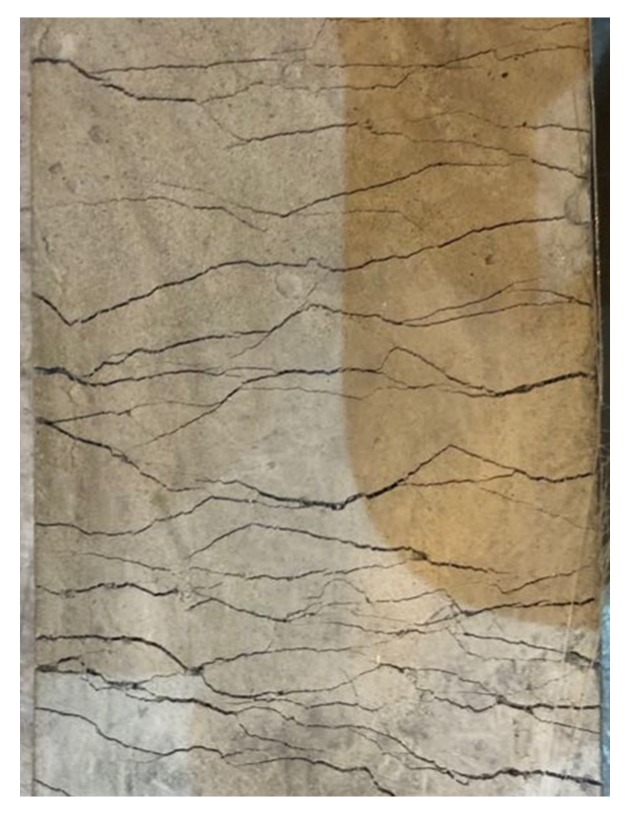
Multiple cracks on HVFA-SHCC specimens.

**Figure 7:**
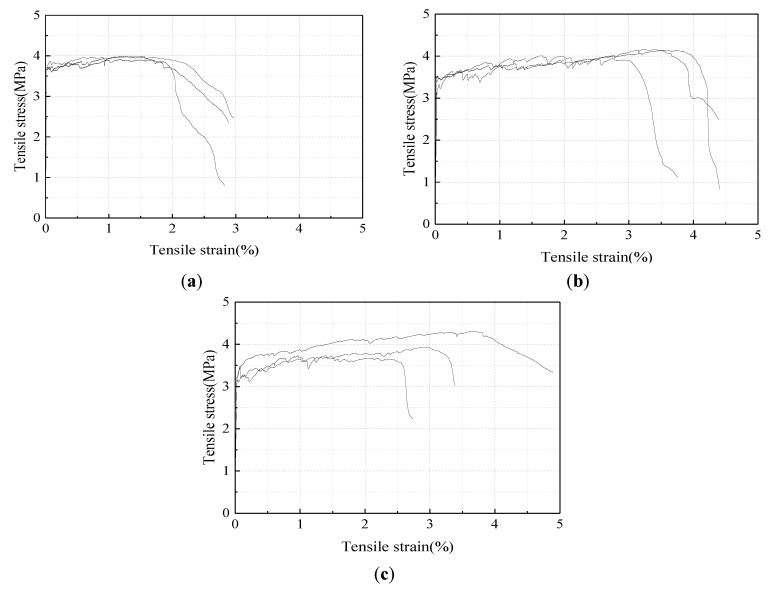
Measured stress-strain curves of HVFA-SHCC samples. (**a**) FA2.4; (**b**) FA3.2; (**c**) FA4.4.

The alignment of the specimens greatly influenced the direct tensile test results. Misalignment of the specimen could lead to eccentric loading in the specimen, and, consequently, the crack width was not uniform along the crack plane. Therefore, multiple cracking could not be fully formed in the misaligned specimens. In contrast, with increasing tension, well aligned specimens exhibited multiple parallel cracks distributed along the specimens during strain hardening, as shown in Figure 6. The tensile stress-strain curves of the three groups of specimens are shown in Figure 7, in which the results of the specimens with obvious misalignment were excluded. It can be seen from the figures that the strain capacity of FA2.4 was obviously lower than those of FA3.2 and FA4.4. The tensile stress-strain curves of FA4.4 specimens were less consistent compared with the curves of the FA3.2 specimens. The average values of measured first crack strength *σ_fc_*, strain *ε_fc_*, peak stress *σ_tp_*, and ultimate tensile strain *ε_tu_* are tabulated in Table 5. It can be seen from the table that the first crack strength decreased with the increase of fly ash content. This is because the increase of fly ash generally leads to a reduction of the early strength. The strain capacity of the FA3.2 specimens (about 3.42%) was significantly larger than that of FA2.4 (about 1.92%). This demonstrates that an increase of fly ash reduced the bond of the fiber–matrix interface and the matrix fracture toughness as well, which rendered the energy criterion for strain hardening more easily satisfied. The average strain capacity of FA4.4 specimens reached 3.07%, but the test results showed more variation, indicating that excessive fly ash content may reduce the stability of the mix.

### 3.3. Uniaxial Compressive Test

#### 3.3.1. Test Preparation for Uniaxial Compressive Test

Compressive strength and elastic modulus are basic and important parameters for construction materials. Six groups of prism specimens (100 mm × 100 mm × 300 mm) with different mixes (FA2.4–FA4.4) were prepared to obtain the compressive strength and elastic modulus of HVFA-SHCCs at 28 days. Meanwhile, mix FA3.2 was selected to investigate the compressive behavior at different ages (7 days, 28 days, and 90 days). At least six specimens were prepared for each mix, three samples for measuring compressive strength and three samples for measuring the elastic modulus of the HVFA-SHCCs. All specimens were tested after curing for the scheduled days in standard curing room.

To measure the compressive strength, the prepared specimens were first pre-loaded to 5 MPa in compression three times, and then tested by a displacement control scheme in a 3000 kN capacity hydraulic servo loading system with a loading rate of 0.3 mm/min. The elastic modulus of the HVFA-SHCC specimens was measured using the same test instrument, but a force control system was applied with a loading rate of 0.3 MPa/s, and the specimens were loaded to stress of about 1/3 of the compressive strength to evaluate the elastic modulus, according to the Chinese standard (GB/T 50081-2002) [38] for testing ordinary concrete. The strain of each specimen was measured with two gauges placed at both sides of each specimen, and determined by the average value of the strain readings of the two gauges.

#### 3.3.2. Test Results of Uniaxial Compressive Test

The specimens were almost linearly elastic before the peak stress was approached. A sudden drop of load was then detected after reaching their compressive strength. At the late phase of loading, several cracks were formed parallel to the loading direction, while transverse deformation of the specimens became obvious. However, unlike ordinary concrete with brittle failure, when the specimens failed, no major spalling of the matrix were observed due to the fiber-bridging action, and the failed specimens could still retain integrity without falling apart.

**Figure 8:**
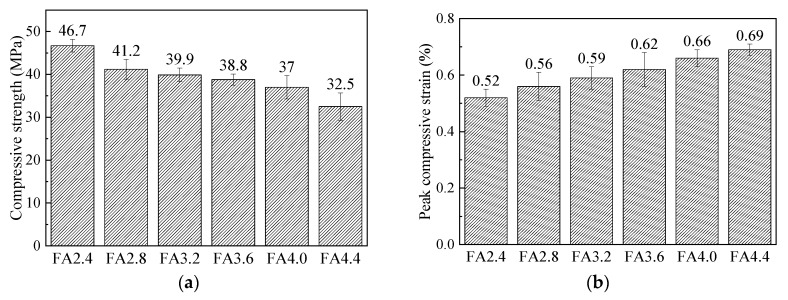

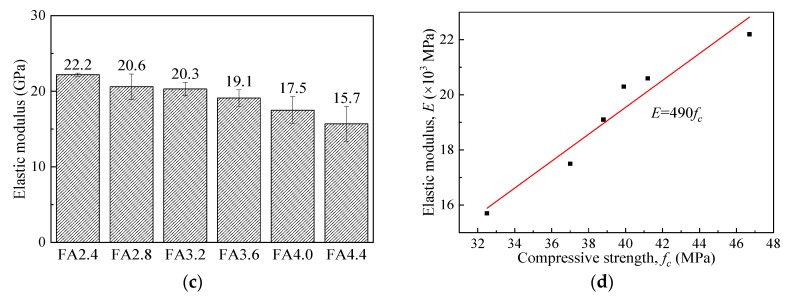
Comparison of the compressive behavior of the mixes. (**a**) Compressive strength; (**b**) peak compressive strain; (**c**) elastic modulus; (**d**) correlation between compressive strength and elastic modulus.

**Figure 9:**
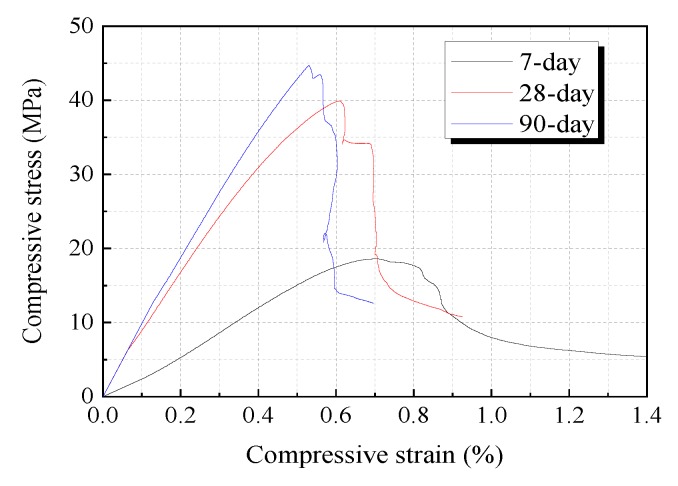
Compressive stress-strain curves of FA3.2 specimens at different ages.

The compressive strength, compressive strain corresponding to the peak stress, and elastic modulus of axial compression at 28 days of each mix are compared in Figure 8. As can be seen from the figures, the average compressive strength at 28 days of HVFA-SHCC specimens with all mixes exceeded 30 MPa, which is suitable for structural applications, and the compressive strength of the HVFA-SHCCs obviously decreased with the increase of FA to cement ratio. The compressive strains at peak stress of tested HVFA-SHCC specimens varied from about 0.5% to 0.7%, which is significantly higher than those of ordinary concrete of a similar grade, and gradually increased with increasing fly ash content. Similarly, the figure also shows that the elastic modulus of the HVFA-SHCCs gradually decreased with the increase of fly ash content, as anticipated, and was generally lower than that of regular structural concrete due to the lack of coarse aggregate. Lower stiffness of the composite can bring advantages in structural applications; for instance, the stress level can be reduced under thermal loadings, but it also has many disadvantages. For example, the control of deflections will be more difficult, and it is also not beneficial to seismic resistance. Therefore, further study could focus on the improvement of elastic modulus to meet different kinds of application requirements. Figure 8d shows the correlation between the compressive strength and elastic modulus of the developed HVFA-SHCCs. The plot indicates that the elastic modulus of the HVFA-SHCCs was almost linear with the compressive strength. A simple fitting line is also shown in the figure. Figure 9 shows the compressive stress-strain curves of the HVFA-SHCC specimens of mix FA3.2 at the ages of 7, 28, and 90 days. It can be seen from the figure that the strength of the FA3.2 specimens developed slowly in the early stage, and the 7 day strength was only 46.6% of that at the age of 28 days. The strength of the specimens then increased rapidly from 7 days to 28 days, and still showed an increase of about 12% at 90 days compared with the results at 28 days. This is because fly ash is relatively inactive, and most of the fly ash particles mainly act as filler, making the hydration slow at the early stage, which leads to a lower compressive strength at the early stage. However, as the age increases, the hydration is increasingly adequate, and fly ash later becomes favorable in the development of strength for HVFA-SHCCs.

### 3.4. Four-Point Bending Test

#### 3.4.1. Test Preparation for Four-Point Bending Test

The multiple cracking behavior of SHCC allows large deflection of samples under bending, which results in the phenomenon known as “bendable concrete”. The four-point bending test can visually exhibit the characteristics of multiple cracking, strain hardening, and high ductility of SHCC materials.

**Figure 10:**
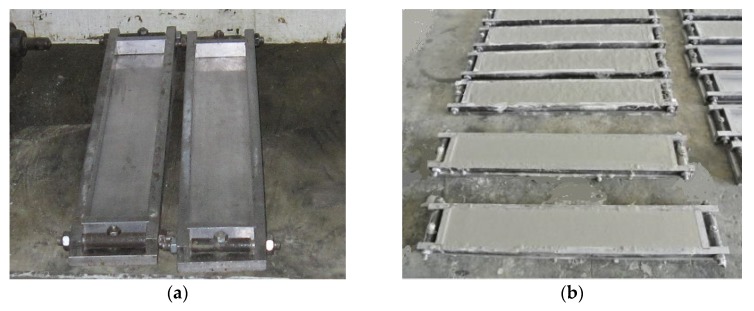
Molds and samples for four-point bending test. (**a**) Coupon molds; (**b**) molded specimens.

**Figure 11:**
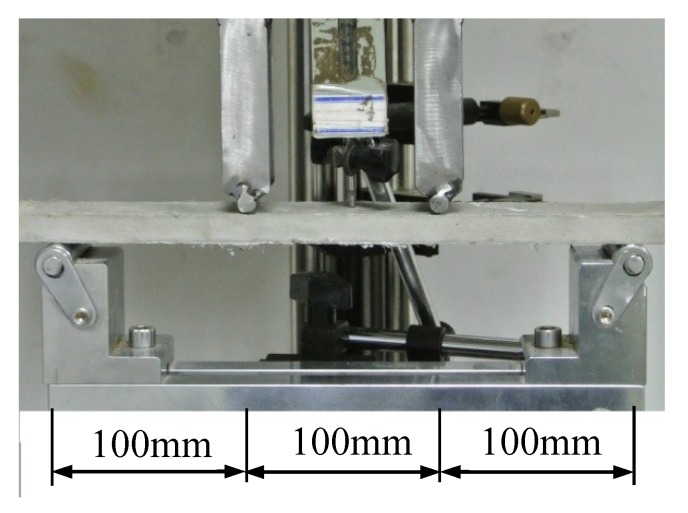
Setup (bending test system, Xinsansi, Shenzhen, China) of four-point bending test.

Coupon specimens were used in the test, with dimensions of 350 mm × 50 mm × 15 mm (length × width × depth). The designed removable steel molds and molded samples are shown in Figure 10. The HVFA-SHCC materials prepared for the four-point bending test were in the same batch as those used in the aforementioned uniaxial compression test. These specimens were also cured in the same environment as the specimens for the tensile and compressive tests. There were at least six specimens for each group. Each specimen was supported by a steel beam with two roller supporters, and the span between two loading points was equal to the shear span between the external support and the closer loading point, as shown in Figure 11. Four-point bending tests were conducted with a hydraulic servo loading system, and displacement control with a loading rate of 0.5 mm/min was applied in the test. A LVDT was placed at the midspan of the specimen to measure its deflection.

#### 3.4.2. Test Results of Four-Point Bending Test

**Figure 12:**
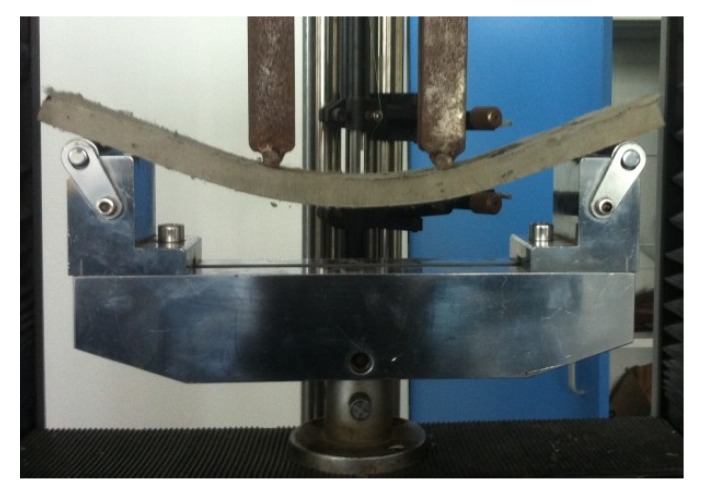
Typical deformation of specimen under bending.

**Figure 13:**
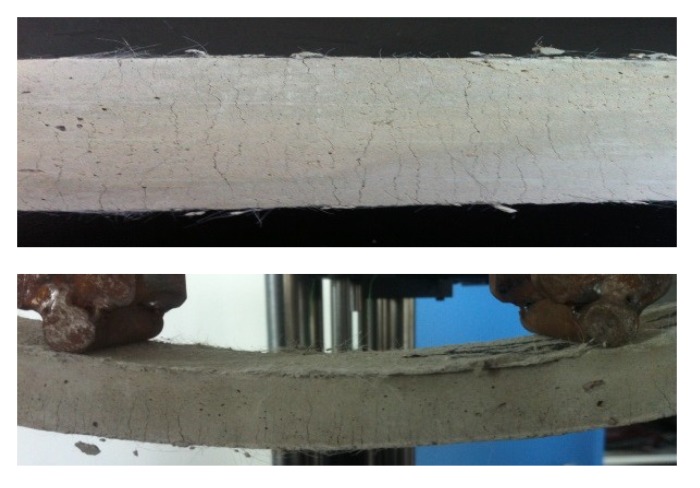
Typical multiple cracking of specimens.

During the testing, all specimens were initially in an elastic state, and the deflection increased linearly with increasing external loading at the early stage. The first crack then generally developed at the biggest flaw within the pure bending region. With increasing displacement, the existing cracks continued to propagate upwards but the crack width did not grow significantly. Meanwhile, multiple fine cracks parallel to the initial cracks developed throughout the whole pure bending region. At this stage, the measured load increased with increasing displacement and fluctuated with the occurrence of newly developed cracks, which exhibited obvious strain-hardening and multiple cracking behavior. After that, the fiber-bridging action in one crack plane could no longer sustain the increasing tensile stresses caused by the moment due to the pulling-out and rupture of fibers, resulting in a dominant crack with an unstable opening, and the load started to decrease until the failure of the specimen. The typical deformation and multiple cracking of the tested HVFA-SHCC specimens are shown in Figure 12 and Figure 13, respectively.

**Figure 14:**
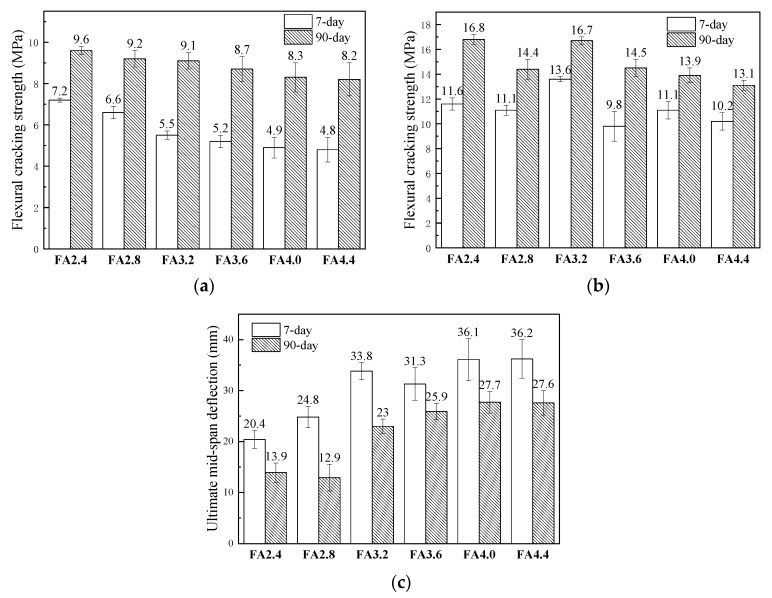
Comparison of bending behavior of specimens with different mixes and ages. (**a**) Flexural cracking strength; (**b**) flexural strength; (**c**) ultimate mid-span deflection.

The comparisons of the flexural behaviors of HVFA-SHCC specimens with different FA to cement ratios and ages (7 days and 90 days) are shown in Figure 14. The bending strength *σ_f_* was calculated according to the following formula:

(4) $$\sigma_{f}=\frac{FL}{bh^{2}}$$

where *F* is the external loading recorded by the test machine; *L* is the span between two supports; *b* is the sectional width of specimens; and *h* is the sectional depth of specimens.

Figure 14a shows that flexural cracking strength of the specimens almost linearly decreased with the increase of fly ash content, indicating that the addition of fly ash reduced the early strength of the matrix. Comparison of the flexural cracking strengths of the specimens at 90 days demonstrates that the strength also decreased with the increase of fly ash content, but the decrease rate was lower than that of the 7 day specimens. This phenomenon indicates that the development of matrix strength grew faster due to more adequate reaction of fly ash. However, the flexural strength of the HVFA-SHCC specimens did not absolutely decrease with the increase of fly ash content, as shown in Figure 14b. The FA3.2 specimens showed the highest 7 day flexural strength, and their 90 day flexural strength was close to that of the FA2.8 specimens and higher than others. This is because the flexural strength was not only influenced by the matrix strength, but also affected by the deformation ability. Although the matrix strength of FA3.2 specimens was lower than those of the FA2.4 and FA2.8 specimens, the higher deformation capacity of the FA3.2 specimens extended their strain-hardening stage and thereby increased the flexural strength. As shown in Figure 14c, the ultimate mid-span deflection basically grew with rising content of fly ash, with exceptions of FA2.8 at 7 days and FA3.2 at 28 days. This indicates that the addition of fly ash had basically a positive influence on the flexural ductility. However, it can be seen through the comparison that differences among the flexural ductilities of FA3.6, FA4.0, and FA4.4 specimens were not significant, indicating that the increase of fly ash no longer improved the flexural ductility of the material significantly after reaching a certain value. Therefore, taking a full account of the tensile, compressive, and flexural performance of the specimens, HVFA-SHCC mix FA3.2 (with a fly ash to cement ratio of 3.2 or fly ash to binder ratio of about 76%) is suggested for structural application.

## 4. Conclusions

A type of sustainable SHCC with a high volume of fly ash (no less than 70% of the binder material) intended for structural applications was developed in this study. A micromechanics-based analysis and a PSD optimization were conducted as guidance for the mix designing of the HVFA-SHCC to fulfil the objectives of tensile strain-hardening behavior with strain capacity over 2% and target compressive strength of 30 MPa. According to the analyses and experiments of mechanical properties of prepared HVFA-SHCC, the following conclusions can be drawn:

(1) Based on the analysis, a higher volume of fiber can provide more complementary energy, however, excessive fibers may negatively affect the workability and fiber dispersion. Chemical and frictional bonds should not be too strong, otherwise, the complementary energy will be insufficient to meet the energy criterion for SHCC. Properly increasing the water to binder ratio and the fly ash to cement ratio can decrease both the bond strength of the interface and the fracture toughness of the matrix, which is beneficial to the formation of strain hardening and multiple cracking.
(2) Based on the PSD optimization, the standard deviation between PSD curve of the designed mixture and the optimal grading curve calculated from the A&A model was less than 10%, and became more steady when the FA to cement ratio exceeded 2.4. The minimal standard deviation appeared when the sand to binder ratio was around 0.4.
(3) Specimens under direct tension tested in this study exhibited obvious multiple cracking and strain-hardening behavior, if the specimens were well aligned. The measured specimens reached tensile strain capacities near or over 2%. When the FA to cement ratio increased from 2.4 to 3.2, the tensile strain capacity increased by 78%; however, when the FA to cement ratio increased from 3.2 to 4.4, the stability reduced while the tensile strain capacity decreased by 10%.
(4) The 7 day compressive strength and elastic modulus of HVFA-SHCCs were relatively lower than those of normal concrete, and prominently decreased with the increase of FA to cement ratio. For Mix FA3.2, the 7 day strength was only 46.6% of that at the age of 28 days, indicating that the initial strength decreased with the addition of a large amount of fly ash. However, all mixes used in this study could exceed the target compressive strength of 30 MPa at 28 days.
(5) Distributed multiple cracks were observed on the prepared HVFA-SHCC specimens during the four-point tests. The first cracking strengths of HVFA-SHCC specimens linearly decreased with increasing fly ash content, while their flexural strengths did not absolutely decrease with the increase of fly ash content, since the flexural strength was influenced not only by the matrix strength, but also by the deformation ability. The ultimate mid-span deflection initially grew with rising content of fly ash, but the increase of fly ash no longer improved the flexural ductility of the material significantly after the replacement rate of fly ash to cement was over 76%.
(6) After taking a full account of the tensile, compressive, and flexural performance of the specimens, HVFA-SHCC mix FA3.2 (with fly ash to cement ratio of 3.2 or fly ash to binder ratio of about 76%) is suggested for structural application.

## Figures and Tables

**Table 1:** Parameters used in the analysis with fiber-bridging model.

*D_f_* (μm)	*L_f_* (mm)	*E_f_* (GPa)	*V_f_* (%)	*τ*_0_ (MPa)	*G_d_* (J/m^2^)	*f_f_* (MPa)
39	12	22	2.0	1.0	1.5	1092

**Table 2:** Mix proportion of high-volume fly ash strain-hardening cementitious composites (HVFA-SHCC).

Sample	Cement (kg)	FA (kg)	S/B Ratio *	W/B Ratio **	*V_f_* (%)	SP (kg)
FA2.4	1.0	2.4	0.36	0.25	2.0	0.0082
FA2.8	1.0	2.8	0.36	0.25	2.0	0.0062
FA3.2	1.0	3.2	0.36	0.25	2.0	0.0071
FA3.6	1.0	3.6	0.36	0.25	2.0	0.0068
FA4.0	1.0	4.0	0.36	0.25	2.0	0.0047
FA4.4	1.0	4.4	0.36	0.25	2.0	0.0042

* S/B ratio = sand to binder ratio; ** W/B Ratio = water to binder ratio.

**Table 3:** Chemical compositions of cement and fly ash (major constituents).

Materials	SiO_2_ (%)	Al_2_O_3_ (%)	Fe_2_O_3_ (%)	CaO (%)	MgO (%)	K_2_O (%)	Na_2_O (%)	SO_3_ (%)	LOI (%)
Fly ash	49.29	27.8	6.63	7.22	0.84	1.21	0.45	0.71	3.99
Cement	20.35	4.38	3.37	63.85	2.13	0.38	0.11	4.26	1.17

**Table 4:** Mechanical properties of PVA fiber.

Type	Diameter (μm)	Length (mm)	Elongation (%)	Density (g/cm^3^)	Elastic Modulus (MPa)	Nominal Strength (MPa)
REC-15	39	12	7	1.3	42.8	1620

**Table 5:** Direct tensile test results.

Sample	*ε_fc_* (%)	*σ_fc_* (MPa)	*ε_tu_* (%)	*σ_tp_* (MPa)
FA2.4	0.019	3.67	1.92	3.97
FA3.2	0.020	3.34	3.42	4.10
FA4.4	0.022	3.26	3.07	3.99
